# Supplementary material for: Biodegradable Nonwovens with Poultry Feather Addition as a Method for Recycling and Waste Management
Source: Polymers (Basel). 2022 Jun 11;14(12):2370. doi: 10.3390/polym14122370 (PMC9230047; doi:10.3390/polym14122370)
Supplement: Supplementary file 1 [file polymers-14-02370-s001.zip › polymers-1700170-supplementary.pdf]

Table S1. SEM photodocementaion of samples

group	nonwoven	SEM<br>100x	SEM<br>500x
I	nonwoven I<br>”0”	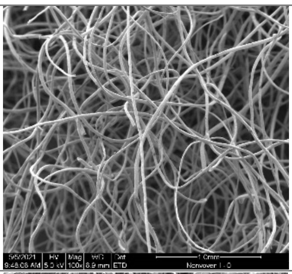	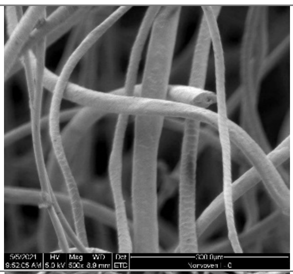
	nonwoven I	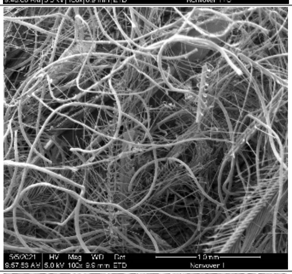	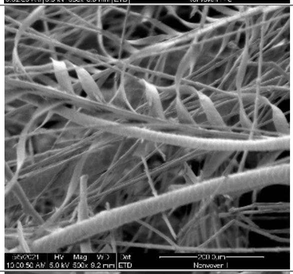
	nonwoven II<br>”0”	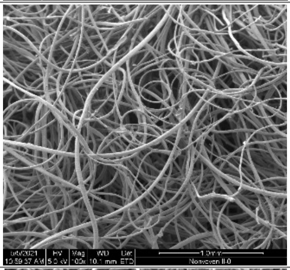	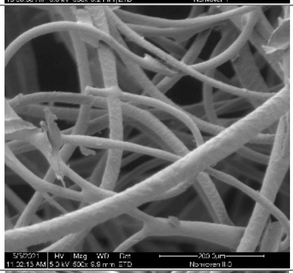
	nonwoven II	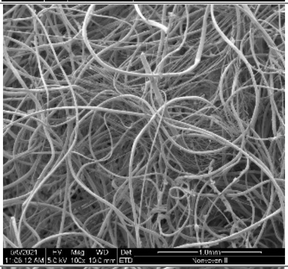	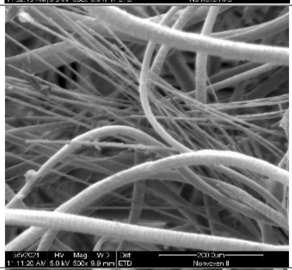
	nonwoven III<br>”0”	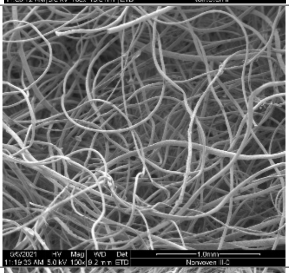	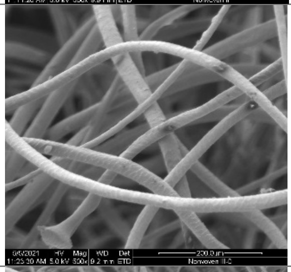
	nonwoven III	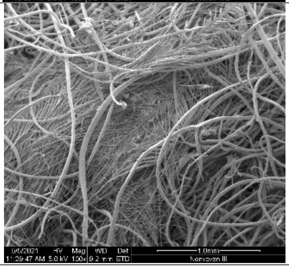	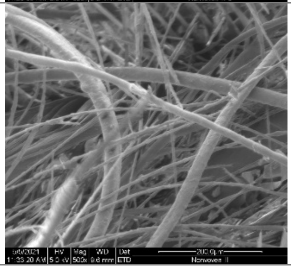

**II**	DA I/1	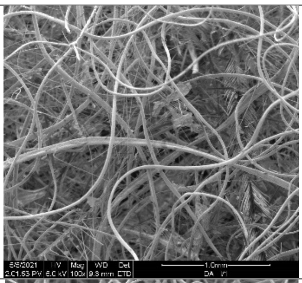	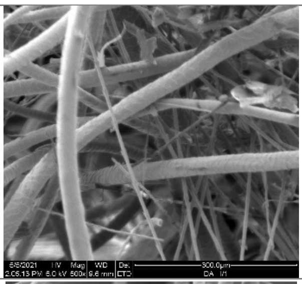
	DA „0" I/1	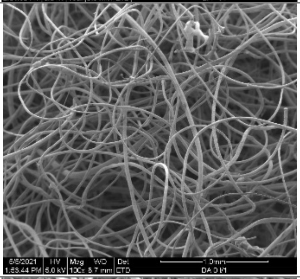	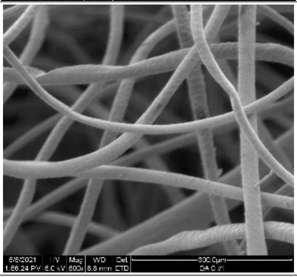
	DA I 1i2	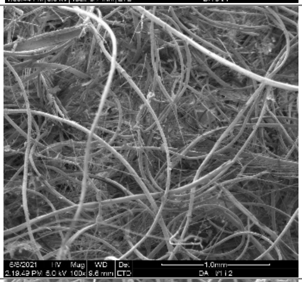	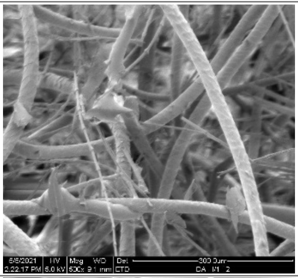
	DA „0" I/1and2	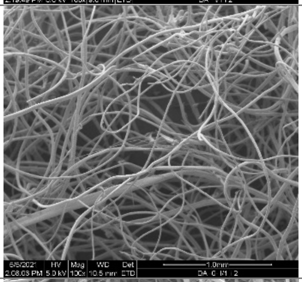	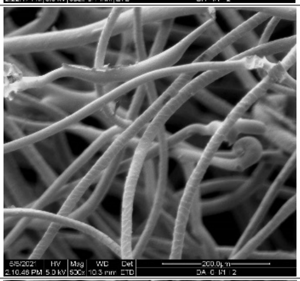
	DA I/2	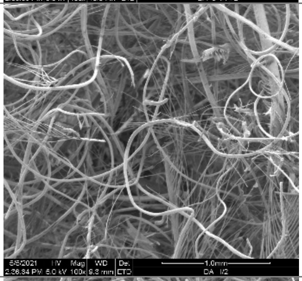	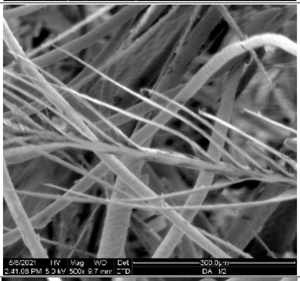
	DA „0" I/2	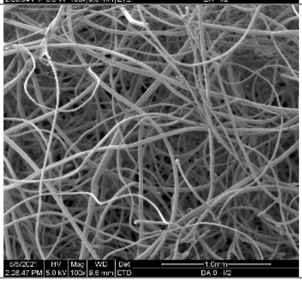	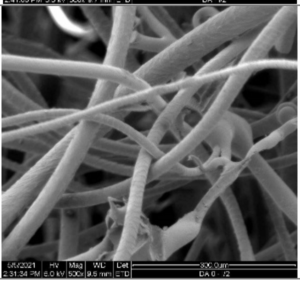

DA II/2	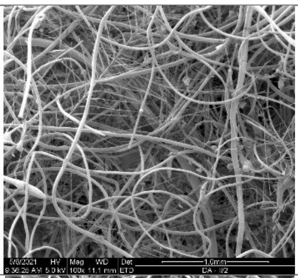	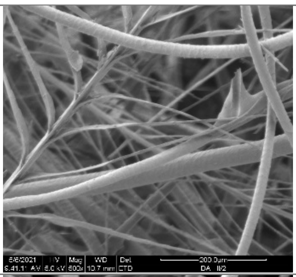
DA „0” II/2	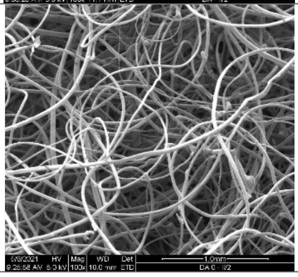	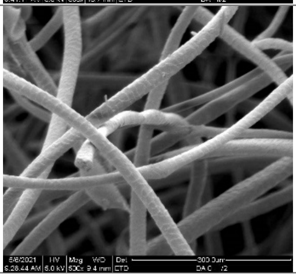
